# Loneliness and its Determinants in Mothers Raising Infants in Central Tokyo

**DOI:** 10.31662/jmaj.2025-0156

**Published:** 2025-11-14

**Authors:** Emilie Louise Akiko Matsumoto-Takahashi, Yuko Matsuoka, Toshiko Eto

**Affiliations:** 1 Division of Global Health, Graduate School of Public Health, St. Luke’s International University, Tokyo, Japan; 2 Department of Tropical Medicine and Malaria, Research Institute, National Center for Global Health and Medicine (NCGM), Tokyo, Japan; 3 Junguri Project, Tokyo, Japan

**Keywords:** loneliness, maternal health, UCLA loneliness scale, Tokyo, Japan

## Abstract

**Introduction:**

In Japan, suicide is the most common cause of death among women who die in the first year after childbirth. This study aimed to analyze the loneliness of mothers raising infants in central Tokyo and to provide evidence for improving maternal mental health.

**Methods:**

Fieldwork was conducted from June to September 2024, and questionnaire surveys were administered to 104 mothers who visited childcare support groups in Setagaya ward, Tokyo. Survey items were socioeconomic indicators and the Japanese version of the University of California, Los Angeles (UCLA) loneliness Scale Version 3 (UCLA-LS3-J), containing 20 items. Structured equation modeling (SEM) was conducted to analyze differences in loneliness by mother and infant characteristics.

**Results:**

The average age of the mothers was 34.4 years, and that of their youngest child was 9.8 months. Among them, 24.0% of the mothers had a UCLA-LS3-J score >40 (which is considered moderate to high in terms of loneliness). SEM identified three factors independently associated with loneliness: health status, daily use of a nursery school or kindergarten, and the number of people the mothers could consult (*p* <0.05). Specifically, loneliness of the mothers was significantly higher when they were feeling less healthy, not using a nursery school or kindergarten, and having fewer people that they could consult.

**Conclusions:**

Some mothers felt particularly isolated, and an intervention to strengthen consultation could be effective in improving loneliness of the mothers living in central Tokyo. Moreover, promotion of the use of nursery schools or kindergartens is also expected to improve the mothers’ mental health.

## Introduction

In Japan, suicide is the most common cause of death among women who die in the first year after childbirth ^(1), (2), (3)^. Although statistics on the number of suicides are available in Japan, until 2022, there were no separate statistics published on the number of suicides committed by pregnant or postpartum women within a year of delivery. In 2017, an examination of abnormal maternal deaths in Tokyo’s 23 wards found 89 abnormal deaths (including 63 suicides) in women during pregnancy or within a year after childbirth from 2005 to 2014 ^(1)^. Specifically, the suicide rate actually reached 8.7 per 100,000 childbirths, which is higher than that in any other country. Furthermore, from 2022, the National Police Agency in Japan started to add new breakdowns for female suicides depending on when they took their own lives (during pregnancy, within 2 months after giving birth, within 3 to 12 months after delivery, and not applicable) ^(3)^. As a result, in 2022, 65 women had committed suicide during pregnancy or within 1 year after giving birth (suicide rate: 8.4 per 100,000 childbirths). Among them, 70% of the suicides took place in the postpartum period.

The progress made in perinatal care in Japan in recent decades has led to a decline in the rates of perinatal, neonatal, and maternal mortality, which are among the lowest in the world ^(4)^. However, while there are fewer childbirths, it has become clear that some mothers take their own lives after successfully giving birth, which is an urgent public health issue that must be resolved. Especially in urban areas of Japan, pregnancy, childbirth, and child-rearing are increasingly completed within the family unit ^(5)^. With the new coronavirus disease of 2019 (COVID-19) pandemic and increasingly weaker regional connections, more and more mothers are being forced to raise their children in isolation, leading to their loneliness. Loneliness is known to be correlated with suicide, and this correlation could assist in the identification of suicidal individuals or those at elevated risk of suicidal behavior ^(6), (7), (8)^. In addition to loneliness, anxiety about child-rearing and changes in their living environment threaten to produce symptoms such as postpartum depression, sleep disorders, eating disorders, and even suicidal thoughts.

Becoming a parent is a major life transition and is generally associated with loneliness. A scoping review on loneliness experienced during pregnancy and the first 5 years of parenthood prior to the COVID-19 pandemic, found that studies were limited, but loneliness was commonly experienced alongside parenting difficulties, with parents feeling as though they were alone in their struggle ^(9)^. Across these literature, age ^(10), (11)^, poor health ^(5), (12)^, friendships ^(13), (14)^, and breast feeding and bottle-feeding ^(14)^ were identified as sources of mother’s loneliness. During the COVID-19 pandemic, a study in married Japanese women found negative consequences for both mental and physical health, as well as elevated severe psychological distress and suicidal ideation ^(15)^. Although findings from previous studies are limited, it is a serious situation that requires immediate improvement and should not be overlooked by society. Therefore, the objectives of this study were to analyze the loneliness of mothers raising infants in central Tokyo. The novelty and originality of the present study is that it provides evidence for improving maternal mental health in this area where knowledge is scarce but of great importance.

## Materials and Methods

### Study design and data collection

We conducted this cross-sectional study from June to September 2024 and analyzed loneliness and its determinants in 104 mothers raising infants living in Setagaya ward, Tokyo. Setagaya is located in the southwestern corner of Tokyo’s special wards and has the largest population (estimated population of 918,141 as of January 2024) and the second largest area among Tokyo’s special wards (58.06 km^2^) ^(16)^. Setagaya has one of the largest juvenile populations, with the average age of the mother at the birth of her first child being around 33 years old, which is older than the national average ^(17)^.

A self-administered, anonymous questionnaire survey was conducted with mothers who visited seven childcare support groups in Setagaya. In Setagaya, these groups are active, and many mothers participate in their activities, thus sufficiently ensuring the representativeness and generalizability of the present study. All the mothers who met the criteria (mothers living in Setagaya ward whose youngest child is 4 years old or younger) were asked and all joined the study. After obtaining appropriate consent from the mothers to cooperate in the research, the questionnaire was distributed to those who agreed to participate. The survey items were selected based on limited previous literature findings ^(5), (9), (10), (11), (12), (13), (14), (15)^ as well as discussions with childcare support groups in Setagaya wards. It included socioeconomic indicators (mother’s age, age of the child, number of children, family structure, living situation, mother’s educational background, mother’s occupation, and family financial situation), mother’s sense of health (four scales: “healthy,” “fairly healthy,” “not very healthy,” and “not healthy”), mother’s ease in raising children, availability of childcare, and the Japanese version of University of California, Los Angeles Loneliness Scale Version 3 (UCLA-LS3-J) score, containing 20 items ^(18), (19)^. The Japanese version of the scale also has a strong reliability and validity (M2), to measure the subjective experience of loneliness by asking participants to rate how often they experience certain feelings related to isolation on a 4-point Likert scale ^(18)^. Confirmatory factor analysis identified that a model incorporating “a global bipolar loneliness factor” and two “method factors” reflecting the direction of item wording provided a very good fit to the data across different samples ^(18)^.

### Statistical analysis

First, a descriptive analysis was conducted to gain an overview of the participants. Second, structured equation modeling (SEM) was used to identify the factors associated with the mother’s loneliness. The correlations of all variables were examined, and a path model was constructed based on the results of a bivariate analysis. The fit of the model was examined in terms of degree of freedom (df), chi-square (CMIN), comparative fit index (CFI), and root mean square error of approximation (RMSEA). According to conventional criteria, a good fit was defined as CMIN/df <2, CFI >0.97, and RMSEA <0.05, and an acceptable fit was defined as CMIN/df <3, CFI >0.95, and RMSEA <0.06 ^(20)^. A *p*-value of <0.05 was considered to indicate statistical significance. All statistical analyses were performed using Stata version 18 and SPSS version 27.0 and Amos 27.0 (SPSS Inc., Chicago, IL, USA).

### Ethical considerations

The present study was approved by the Ethical Committee of St. Luke’s International University (approval number 24-R062). The researchers obtained informed consent from all participants of the survey.

## Results

### Participants

The survey was conducted in seven sites, and 104 mothers participated in the study (Table 1). Eighteen of the present participants visited these sites for the first time. Among all mothers, 87 (83.7%) had one child, and the rest had more than two children. The average age of the mothers was 34.4 years old, and for most, their youngest child was younger than 2 years old. Most family structures comprised a nuclear family (couple and children), whereas four comprised an extended family (e.g., living with grandparents). Only three mothers were living in their parent’s home, and the others were mostly living in their own home. About half of them had been living in their present home for less than 3 years. Among the participants, 74.0% had a university diploma, and 66.3% were employed. For the health status question, 79 replied that they were healthy, and 25 said they were fairly healthy. Although their economic situation and the ease of raising children varied, most were feeling comfortable or a little comfortable with both of these factors.

**Table 1.** Variables per Loneliness Level According to the UCLA Loneliness Scale Version 3 Score (N = 104).

Variables	Stratification by loneliness level						p-Value	
	Lower (<40) (n = 79)		Higher (≥40) (n = 25)		Overall (N = 104)			
	n	%	n	%	n	%		
Data collection location							0.506	^1^
Site 1	2	2.5	0	0.0	2	1.9		
Site 2	4	5.1	1	4.0	5	4.8		
Site 3	26	32.9	7	28.0	33	31.7		
Site 4	36	45.6	10	40.0	46	44.2		
Site 5	2	2.5	1	4.0	3	2.9		
Site 6	0	0.0	1	4.0	1	1.0		
Site 7	9	11.4	5	20.0	14	13.5		
First visit							0.498	^1^
Yes	15	19.0	4	16.0	19	18.3		
No	64	81.0	21	84.0	85	81.7		
Number of children						0.0	0.307	^1^
1	66	83.5	21	84.0	87	83.7		
2	12	15.2	3	12.0	15	14.4		
3	0	0.0	1	4.0	1	1.0		
4	1	1.3	0	0.0	1	1.0		
Mother’s age (years)							0.033	^2^
Mean	33.8	-	36.1	-	34.4	-		
SD	4.4	-	5	-	4.6	-		
Age of the youngest child (years)							0.167	^1^
0	32	40.5	13	52.0	45	43.3		
1	27	34.2	5	20.0	32	30.8		
2	4	5.1	0	0.0	4	3.8		
3	2	2.5	1	4.0	3	2.9		
4	0	0.0	1	4.0	1	1.0		
Age of the next youngest child (years)				0.0			0.788	^1^
2	3	3.8	0	0.0	3	2.9		
3	3	3.8	2	8.0	5	4.8		
4	2	2.5	1	4.0	3	2.9		
5	3	3.8	1	4.0	4	3.8		
6	2	2.5	0	0.0	2	1.9		
Family structure							0.042	^1^
Nuclear family	78	98.7	22	88.0	100	96.2		
Extended family	1	1.3	3	12.0	4	3.8		
Single parent	0	0.0	0	0.0	0	0.0		
Residence status							<0.001	^1^
Parent’s home	0	0.0	3	12.0	3	2.9		
Home	77	97.5	18	72.0	95	91.3		
Other	2	2.5	4	16.0	6	5.8		
Number of years at current residence		0.0		0.0			0.06	^1^
Less than 1 year	13	16.5	1	4.0	14	13.5		
1 to 3 years	23	29.1	4	16.0	27	26.0		
3 to 5 years	8	10.1	6	24.0	14	13.5		
5 years or more	14	17.7	2	8.0	16	15.4		
Final education							0.06	^1^
High school diploma	3	3.8	1	4.0	4	3.8		
Vocational school diploma	19	24.1	1	4.0	20	19.2		
Junior college diploma	1	1.3	2	8.0	3	2.9		
University diploma or higher	56	70.9	21	84.0	77	74.0		
Occupation							0.566	^1^
Employed	50	63.3	19	76.0	69	66.3		
Self-employed	4	5.1	2	8.0	6	5.8		
Part time job	4	5.1	1	4.0	5	4.8		
Housewife	18	22.8	2	8.0	20	19.2		
Other	3	3.8	1	4.0	4	3.8		
Health status							0.014	^1^
Healthy	65	82.3	14	56.0	79	76.0		
Fairly healthy	14	17.7	11	44.0	25	24.0		
Economic situation							0.779	^1^
Comfortable	14	17.7	4	16.0	18	17.3		
A little comfortable	55	69.6	19	76.0	74	71.2		
A little suffering	10	12.7	2	8.0	12	11.5		
Ease of raising children							0.118	^1^
Comfortable	21	26.6	5	20.0	26	25.0		
Somewhat comfortable	51	64.6	15	60.0	66	63.5		
Somewhat uncomfortable	4	5.1	5	20.0	9	8.7		
Uncomfortable	2	2.5	0	0.0	2	1.9		
Number of people who see your child on a daily basis								
Mean	1.62	-	1.28	-	1.54	-	0.247	^2^
SD	1.27	-	1.27	-	1.28	-		
People who can see your child on daily basis								
Partner	60	75.9	17	68.0	77	74.0	0.43	^1^
Own mother	28	35.4	5	20.0	33	31.7	0.225	^1^
Mother-in-law	4	5.1	2	8.0	6	5.8	0.389	^1^
Relatives	0	0.0	0	0.0	0	0.0	-	^1^
Siblings	4	5.1	0	0.0	4	3.8	0.21	^1^
Nursery school and kindergarten teachers	19	24.1	1	4.0	20	19.2	0.043	^1^
Friends	18	22.8	1	4.0	19	18.3	0.033	^1^
Childcare companion	0	0.0	0	0.0	0	0.0	-	^1^
Other	2	2.5	1	4.0	3	2.9	0.412	^1^
Number of people who can see your child in an emergency								
Mean	1.85	-	1.56	-	1.78	-	0.297	^2^
SD	1.25	-	1	-	1.2	-
People who can see your child in an emergency								
Partner	60	75.9	17	68.0	77	74.0	0.442	^1^
Own mother	41	51.9	12	48.0	53	51.0	0.82	^1^
Mother-in-law	17	21.5	7	28.0	24	23.1	0.587	^1^
Relatives	4	5.1	1	4.0	5	4.8	1	^1^
Siblings	9	11.4	0	0.0	9	8.7	0.11	^1^
Nursery school and kindergarten teachers	7	8.9	2	8.0	9	8.7	1	^1^
Friends	5	6.3	0	0.0	5	4.8	0.334	^1^
Childcare Companion	0	0.0	0	0.0	0	0.0	-	^1^
Other	3	3.8	0	0.0	3	2.9	1	^1^
Number of places where they meet								
Mean	1.19	-	1.08	-	1.16	-	0.326	^2^
SD	0.53	-	0.28	-	0.49	-
Types of places they meet								
Child-rearing salons	11	13.9	1	4.0	12	11.5	0.285	^1^
Other (face-to-face)	5	6.3	1	4.0	6	5.8	1	^1^
Other (online)	3	3.8	0	0.0	3	2.9	1	^1^
None	3	3.8	1	4.0	4	3.8	1	^1^
Numbers of types of care support used								
Mean	0.9	1.1	0.92	3.7	0.9	0.9	0.81	^2^
SD	0.41	0.5	0.27	1.1	0.38	0.4		
Types of care support used
Family support	0	0.0	0	0.0	0	0.0	-	^1^
Babysitter	3	3.8	1	4.0	4	3.8	1	^1^
Housework service	3	3.8	0	0.0	3	2.9	1	^1^
Other	7	8.9	1	4.0	8	7.7	0.608	^1^
None	0	0.0	0	0.0	0	0.0	-	^1^
Number of people you can consult								
Mean	3.81	4.8	2.76	11.0	3.56	3.4	0.006	^2^
SD	1.6	2.0	1.69	6.8	1.68	1.6		
People you can consult								
Partner	74	93.7	22	88.0	96	92.3	0.395	^1^
Grandparents	60	75.9	14	56.0	74	71.2	0.076	^1^
Relatives	10	12.7	1	4.0	11	10.6	0.29	^1^
Siblings	24	30.4	4	16.0	28	26.9	0.201	^1^
Friends	58	73.4	12	48.0	70	67.3	0.027	^1^
Acquaintances	5	6.3	1	4.0	6	5.8	1	^1^
Family doctor	16	20.3	2	8.0	18	17.3	0.228	^1^
Nurse	1	1.3	0	0.0	1	1.0	1	^1^
Midwife	10	12.7	5	20.0	15	14.4	0.348	^1^
Healthcare worker	5	6.3	2	8.0	7	6.7	0.673	^1^
Nursery schoolteacher	15	19.0	3	12.0	18	17.3	0.552	^1^
Staff of childcare support facilities	22	27.8	2	8.0	24	23.1	0.055	^1^
Others	1	1.3	1	4.0	2	1.9	0.425	^1^
None	0	0.0	0	0.0	0	0.0	-	^1^

SD: standard deviation; UCLA: University of California, Los Angeles. ^1^Fisher’s exact test. ^2^*t*-test.

Table 1 also shows the number and kinds of people who can see their child on a daily basis and in an emergency, the places they meet, care support use, and people they can consult. Mothers whose children attended nursery school or kindergarten daily had significantly lower loneliness (*p* = 0.043). The number of people they could ask to take care of their child in an emergency, or the number of places where they meet had no significant impact on mother’s loneliness. However, the number of people they could consult significantly reduced their loneliness, and friends and staff at childcare support facilities had a significant role.

### Loneliness of participants

**Figure 1.**
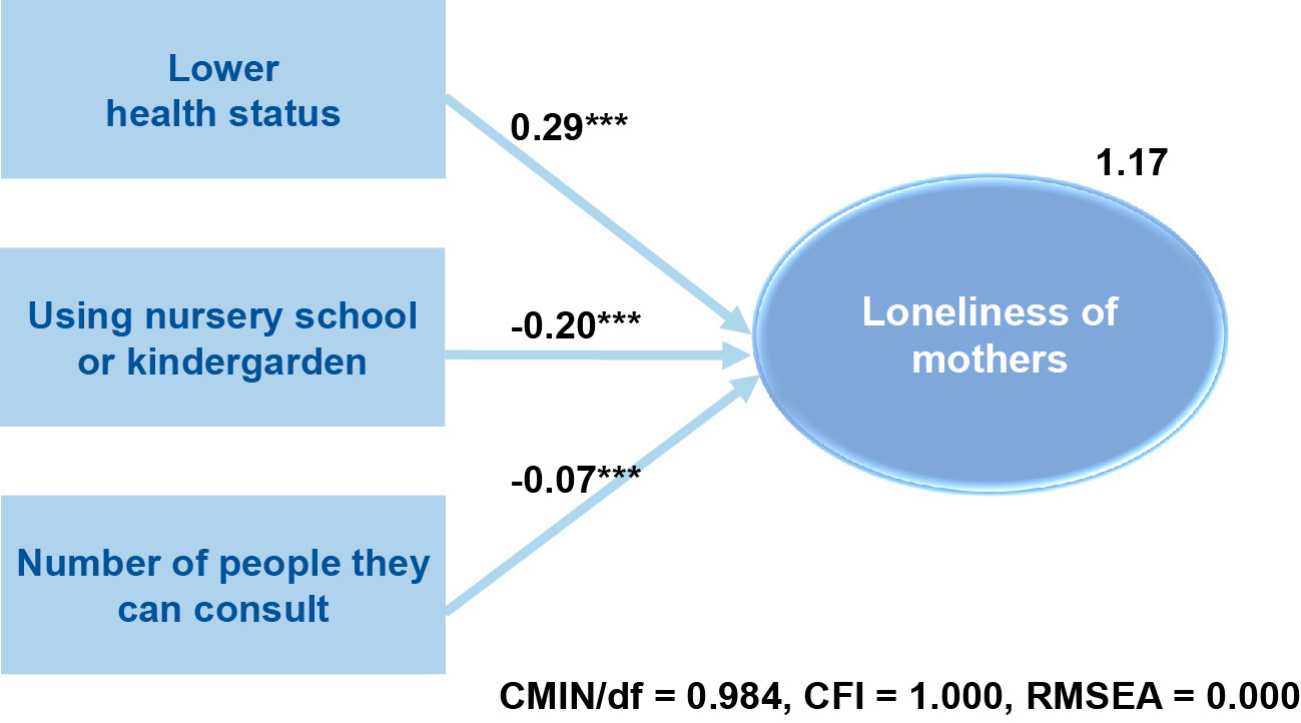
Structural equation model of the loneliness of mothers. CFI: comparative fit index; CMIN: chi-square; df: degree of freedom; RMSEA: root mean square error of approximation.

Among the mothers, 24.0% had a score on the UCLA-LS3-J of >40, which is considered moderate to high in terms of loneliness. Using the results of bivariate analysis and by considering multi-collinearity, SEM was conducted, and the best model with a very good fit (CMIN/df = 0.984, CFI = 1.000, RMSEA = 0.000) is shown in Figure 1. SEM identified three factors that were independently associated with mothers’ loneliness: health status, use of a nursery school or kindergarten on a daily basis, and the number of people the mothers could consult (*p* <0.05). Specifically, loneliness of the mothers was significantly higher when they were feeling less healthy, not using a nursery school or kindergarten, and having fewer people that they could consult.

## Discussion

The objectives of this study were to analyze the loneliness of mothers raising infants in central Tokyo, and to provide evidence for improving their maternal mental health. The study identified three factors that significantly and independently impacted maternal mental health and their loneliness: health status, use of a nursery school or kindergarten on a daily basis, and the number of people the mothers could consult (*p* <0.05).

First, a good health status was significantly associated with less loneliness. All respondents reported health or a little health, and those who reported good health were less lonely than those who reported little health. It is assumed that those who responded little health had a background of hesitation and inability to declare clearly that they were “healthy,” although they did not respond “not healthy.” Loneliness has been associated with ill health and is common in the developed world, and in addition to being a major predictor of psychological problems, some reviews show that loneliness is related to increased risk of chronic conditions, obesity, and poor self-rated health ^(21), (22), (23)^.” There is a strong link between physical and mental health ^(24), (25), (26), (27), (28)^, one study found significant indirect effects explaining 10% of the effect of past mental health on physical health, and 8% of the effect of past physical health on mental health ^(26)^. The key indirect pathways were past cigarette consumption, past physical activity, and past social interaction. Many studies on the relationship between loneliness and health status have focused specifically on the elderly ^(29), (30), (31)^. Both social isolation and loneliness were significantly associated with a greater risk of being inactive, smoking, reporting multiple health-risk behaviors, blood pressure, C-reactive protein level, and even fibrinogen levels. Therefore, improving the loneliness of mothers in central Tokyo is something that has synergy, not only for improving their mental health status, but also for improving their physical health status. Raising a child in the conditions of sleep deprivation and poor health is extremely demanding. Setagaya ward offers temporary childcare (including overnight stays), and its effectiveness should be examined and made available to more mothers.

Second, the daily use of nursery school or kindergarten was also associated with the mothers’ loneliness. There is evidence showing that stay-at-home mothers experience higher levels of depression, parenting anxiety, and parenting stress ^(32), (33)^. Although the relationship between mothers and childcare providers may have some influence, childcare providers have the potential to be good accompanists and collaborators with the mothers. The opportunity to send children to these schools, instead of spending time at home alone with them, will increase the opportunity for mothers to get out of the house and talk with teachers at these schools and with other parents. In addition, mothers will have more time to themselves while their children are in these schools, thus allowing them to calmly assess the current situation, meet friends, and communicate with adults, thereby decreasing their sense of isolation. In some areas, especially in central Tokyo, competition to enroll children, especially in nursery schools, is fierce. Currently, in many cases, only children of working mothers can enroll in daycare centers in Tokyo, but if more facilities were opened for non-working mothers who could casually care for their children, this could be an intervention that might greatly improve the sense of loneliness among mothers.

Third, regardless of the age or education of the mothers, having fewer people that they can consult was a risk factor that increased loneliness. In a survey conducted in Yokohama, Japan’s second largest city, on loneliness among the mothers raising children under 3 years old, as in our present study, the mothers’ loneliness was attributed to the lack of people they could talk to about parenting ^(34)^. Loneliness is the subjective feeling of human experience when people feel less socially connected to others than they desire ^(35), (36), (37)^. Social distancing and isolation during the CoOVID-19pandemic exacerbated the prevalence of loneliness, and supportive consultations were helpful. In the study sample by Hooker et al. ^(35)^, these lonely patients also required greater health care utilization, including longer hospital stays and more primary care visits and referrals. Social networks are known to be positively associated with better mental health outcomes and also to reduce morbidity and mortality ^(38)^.

By creating a system that provides easy access to consultation on child-rearing and other issues, it may be possible to reduce the sense of loneliness among mothers living in central Tokyo. To achieve the above, it will be necessary to clarify how to improve the accessibility of consultation services and strengthen cooperation between supporters, among others. Another determining factor found in the present study was the use of a nursery school, which increases opportunities for casual consultation between teachers and parents. Again, there is a need to improve the capacity of these nursery schools, especially in Tokyo.

Finally, the strength of this study lies in the fact that it collected and carefully analyzed data from mothers in urban centers in Tokyo, Japan, where information is scarce, and obtained interesting results with a good fit, that are conducive to intervention. The limitations of the present study are that the analysis is limited to the central Tokyo area, that it is a cross-sectional study and cannot account for causal relationships, and because of the sampling method and limited sample size, the results are restricted, and the analysis is not broken down by socioeconomic indicators. In Japan, where the maternal and infant mortality rates are very low, the presence of mothers who take their own lives after safely giving birth is a very serious public health issue. This is a topic that requires increased focus, and the present study provided useful information as a starting point.

### Conclusion

Some of the mothers in the present study showed feelings, particularly of isolation, and an intervention to strengthen consultation with them could be effective in improving the loneliness of these mothers living in central Tokyo. Moreover, promotion of the use of nursery schools or kindergartens would also be expected to improve the mothers’ mental health.

## Article Information

### Author Contributions

Emilie Louise Akiko Matsumoto-Takahashi: Writing - review & editing, Writing - original draft, Software, Methodology, Investigation, Funding acquisition, Formal analysis, Conceptualization. Yuko Matsuoka: Writing - review & editing, Methodology, Investigation, Conceptualization. Toshiko Eto: Writing - review & editing, Software, Methodology, Investigation.

### Conflicts of Interest

None

### Availability of Data and Materials

Data are unsuitable for public deposition due to ethical restrictions and the legal framework of Japan. All inquiries about access to data should be sent to the first author.

### Ethical Approval

The present study was approved by the Ethical Committee of St. Luke’s International University (approval number 24-R062). The researchers obtained informed consent from all participants in the survey.

### Consent for Publication

Not applicable.
